# Crossing the Chloride Channel: The Current and Potential Therapeutic Value of the Neuronal K^+^-Cl^−^ Cotransporter KCC2

**DOI:** 10.1155/2019/8941046

**Published:** 2019-05-21

**Authors:** Luke Tillman, Jinwei Zhang

**Affiliations:** Institute of Biomedical and Clinical Sciences, Medical School, College of Medicine and Health, University of Exeter, Hatherly Laboratories, Exeter EX4 4PS, UK

## Abstract

Chloride (Cl^−^) homeostasis is an essential process involved in neuronal signalling and cell survival. Inadequate regulation of intracellular Cl^−^ interferes with synaptic signalling and is implicated in several neurological diseases. The main inhibitory neurotransmitter of the central nervous system is *γ*-aminobutyric acid (GABA). GABA hyperpolarises the membrane potential by activating Cl^−^ permeable GABA_A_ receptor channels (GABA_A_R). This process is reliant on Cl^−^ extruder K^+^-Cl^−^ cotransporter 2 (KCC2), which generates the neuron's inward, hyperpolarising Cl^−^ gradient. KCC2 is encoded by the fifth member of the solute carrier 12 family (*SLC12A5) *and has remained a poorly understood component in the development and severity of many neurological diseases for many years. Recent advancements in next-generation sequencing and specific gene targeting, however, have indicated that loss of KCC2 activity is involved in a number of diseases including epilepsy and schizophrenia. It has also been implicated in neuropathic pain following spinal cord injury. Any variant of* SLC12A5* that negatively regulates the transporter's expression may, therefore, be implicated in neurological disease. A recent whole exome study has discovered several causative mutations in patients with epilepsy. Here, we discuss the implications of KCC2 in neurological disease and consider the evolving evidence for KCC2's potential as a therapeutic target.

## 1. Introduction

Chloride (Cl^−^) is an abundant anion involved in a variety of physiological processes including gene regulation [1, 2], pH maintenance [3], and control of cell volume [4]. Primarily important in the neuron, Cl^−^ plays a crucial role in signalling within the central nervous system (CNS). Healthy brain function requires the correct balance of neuronal excitation and inhibition to determine the firing of action potentials. Action potentials enable rapid propagation of signals. Imbalance of inhibitory and excitatory signals can lead to the development of neurological insults [5–7].

The main inhibitory neurotransmitter, *γ*-aminobutyric acid (GABA), binds to the ionotropic receptor GABA type A channel (GABA_A_R) [8–10]. GABA's role in signalling depends on the intracellular Cl^−^  ([Cl^−^]_i_) concentration, which determines the reversal potential for GABA_A_ currents (*E*_GABA_). *E*_GABA_ lies close to the resting membrane potential (RMP) [11, 12]. Both *E*_GABA_ and RMP vary between cell types and compartments. The depolarising or hyperpolarising effect of GABAergic signalling is dependent on the relative RMP and *E*_GABA_. When [Cl^−^]_i_ is high, *E*_GABA_ is less negative and GABA stimulation results in depolarisation; when it is low, *E*_GABA_ is more negative and GABA stimulation is hyperpolarising [13, 14]. In healthy adult neuron's [Cl^−^]_i_ is usually maintained at a low concentration, enabling inhibitory, hyperpolarising GABAergic signalling [15]. This constitutes the main role of GABA in CNS neurotransmission; its potential dysfunction in neurological disease due to dysregulated cellular Cl^−^ levels is, therefore, of significant interest. Depolarising GABA potentials, in contrast, are commonly observed in immature and peripheral neurons [16]. Finally, in addition to GABA's role in hyperpolarisation, it is able to act in a further inhibitory capacity via the mechanism of “shunting inhibition.” This process involves increased membrane conductance as a result of GABA stimulation “short circuiting” nearby excitatory potentials without producing a significant change in membrane potential.

**Figure 1:**
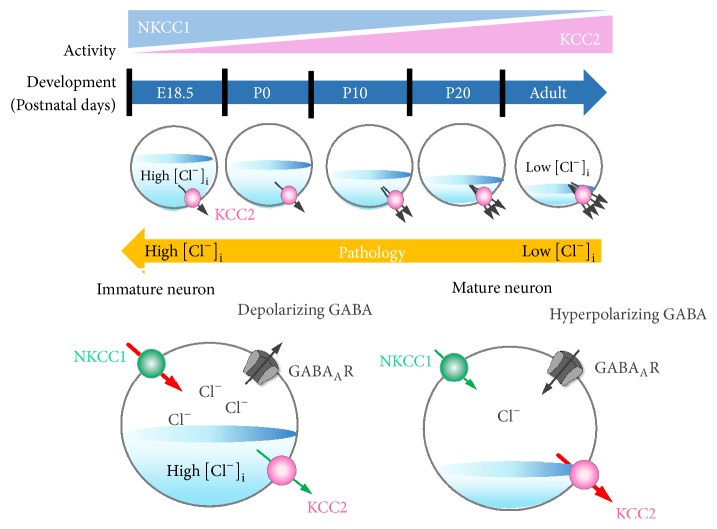
**G** **A** **B** **A**_**A**_ **signalling shifts from depolarizing to hyperpolarising responses during development**. In immature pyramidal cells, GABA_A_R-mediated Cl^−^ currents are outward and depolarising because the relative ratio of NKCC1 to KCC2 activity is high. In mature neurons, increased KCC2 activity gives rise to inward GABA_A_-mediated Cl^−^ currents that hyperpolarize the membrane potential.

**Figure 2:**
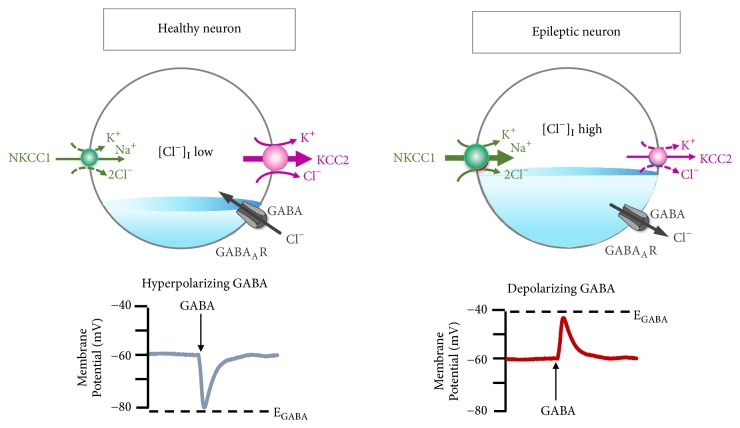
**Neuronal Cl** ^**−**^ **volume is reciprocally regulated via NKCC1 and KCC2**. In healthy mature neurons, [Cl^−^]_i_ is low due to the opposing activity profiles of NKCC1 and KCC2. This promotes GABA_A_R-mediated hyperpolarization, which is critical for the proper balance of excitation-inhibition in neuronal circuits (left panel). In neurons implicated in multiple neuropsychiatric conditions driven by hyperexcitable circuits (e.g., seizures, neuropathic pain, spasticity, schizophrenia, and others), [Cl^−^]_i_ are elevated due to increased NKCC1 activity, and/or decreased KCC2 activity, promoting GABA_A_R-mediated membrane depolarization and excitation (right panel).

Neuronal [Cl^−^]_i_ is regulated by the Na^+^-K^+^-2Cl^−^ cotransporter 1 (NKCC1) and the K^+^-Cl^−^ cotransporter 2 (KCC2) [17]. Using the Na^+^ gradient generated by Na/K/ATPase, NKCC1 drives Cl^−^ into the cell; KCC2, in contrast, is the main Cl^−^ extruder in mature neurons [18]. During development, NKCC1 and KCC2 expression patterns change. In the immature CNS, NKCC1 dominates resulting in high [Cl^−^]_i_. As maturation proceeds, KCC2 expression increases whilst NKCC1 levels fall (Figure 1) [19, 20]. Mature neurons, therefore, have low [Cl^−^]_i_ causing a shift in *E*_GABA_ from depolarising to hyperpolarising [19, 20]. Thus, KCC2 is a crucial regulator of GABA-mediated hyperpolarisation: an essential component of synaptic inhibition within the adult brain (Figure 2).

Loss of KCC2 activity orchestrates a depolarising shift in *E*_GABA_ and is implicated in cortical development systems such as neuro- and synaptogenesis [12, 21]. The fundamental role that KCC2 downregulation plays in these processes suggests a causal link between Cl^−^ homeostasis and the pathogenesis of neurodevelopmental disorders [22, 23]. Although categorised differently, neurodevelopmental disorders including autism and schizophrenia display phenotypic similarities, most notably high copy number variation [24]. These attributes suggest a genetic link between these diseases.

Excitatory and inhibitory imbalance is implicated in the onset of epilepsy. Biopsies of epileptic tissue have identified excitatory GABA activity in response to loss of KCC2 expression and subsequent [Cl^−^]_i_ increase [25–27]. Similarly, in Huntington's disease positive rat models, upregulation of NKCC1 and loss of KCC2 caused GABA mediated stimulation to switch from an inhibitory to excitatory response [28]. Collectively, these studies suggest that researching expression patterns of KCC2 may further our understanding of the aetiology of these diseases.

The aim of this review is to evaluate the role of KCC2 in various pathological conditions. Consideration will first be given to the structure of KCC2; how this affects its function and expression is a key component to understanding its role in disease. Attention will also be given to specific diseases in which KCC2 dysfunction is implicated. Finally, KCC2 will be discussed as a pharmaceutical target for neurological diseases.

## 2. Structure and Diversity of the Cl^−^ Cotransporter KCC2

The KCC2 Cl^−^ cotransporter is transcribed from the fifth member of the solute carrier 12 (*SLC12A5*) gene family. During alternative splicing,* SLC12A5* produces two isoforms: KCC2a and KCC2b [29]. The KCC2a transcript is commonly expressed in the spinal cord between embryonic day (E) 14 and postnatal day (P) 60, whilst KCC2b is greatly upregulated in the hippocampus and the neocortex between E17-P14 [29]. As development progresses, KCC2a expression falls whilst KCC2b is upregulated in the mature CNS. KCC2a is, therefore, the favoured isoform in the immature brain but is eventually dominated by KCC2b in adulthood [30]. Structural differences between these isoforms are localised to the N-terminus where they possess a unique 40 amino acid structure. Despite this, their ion transport activity is almost identical [31]. For the purposes of this review, KCC2 denotes KCC2b.

**Figure 3:**
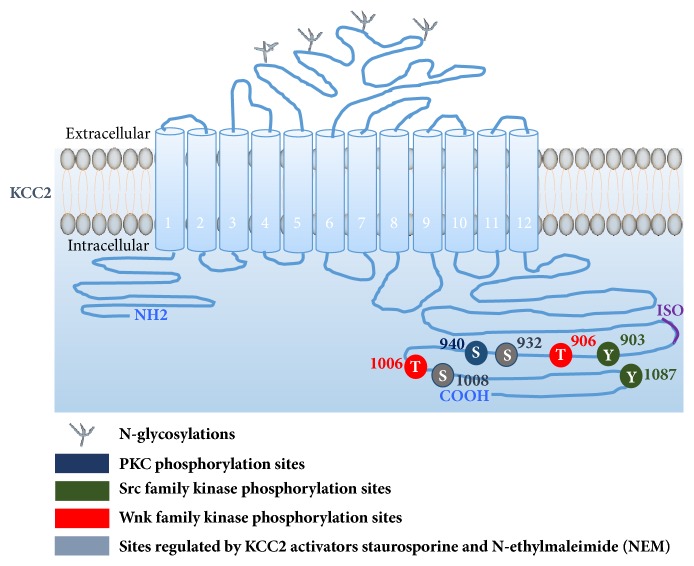
**Schematic representation of important regulatory phosphoresidues of the neuron-specific K–Cl cotransporter KCC2**. The mouse KCC2 co-transporter consists of 12 membrane spanning predicted segments, an N-linked glycosylated extracellular domain between transmembrane domains 5 and 6. This is flanked by two cytoplasmic carboxy- and amino-terminal domains of 104 and 481 amino acids, respectively. Positions of phosphoresidues that are critical for functional regulation of KCC2, including tyrosine 903 (Y903), threonine 906 (T906), serine 940 (S940), threonine 1006 (T1006, this site is corresponding to Rat T1007), tyrosine 1087 (Y1087), and S932 and T1008 (regulated by KCC2 activators staurosporine and N-ethylmalemide (NEM)), are indicated. The Purple region denotes the KCC2 ‘ISO' domain, required for hyperpolarizing GABAergic transmission.

Although KCC2 is one of the most heavily researched transporters within the CNS, limitations in X-ray analysis have led to poor understanding of its structure and functional mechanisms. Hydropathy blot analysis suggests that KCC2 contains 12 transmembrane domains anchored by intracellular N- and C-termini [32]. Precisely half of the protein is intracellular and is the target for a number of kinases and a single phosphatase (Figure 3). Studies have begun to uncover an integral role of the C-terminus in KCC2 activity [33]. For example, posttranslational modifications - phosphorylation and/or glycosylation have been associated with the extrusive qualities displayed by KCC2 [34–36]. During development, KCC2 assembly becomes more complex, with immature brains displaying a higher monomeric count whilst oligomerisation correlates with maturation [37]. More recently, Agez and colleagues showed that KCC2 exists in a monomeric and dimeric state in solution [38]. The same group also noted that peptide C-terminal tagging of KCC2 caused detrimental functional changes and inactivation when expressed in HEK293 cells [38]. Their findings suggest a crucial role of the KCC2 C-terminus in its activity.

Whilst these findings provide insight into the functional significance of KCC2 structure, they fail to show this effect in a neuronal setting. HEK293 are an embryonic kidney cell line commonly used in the analysis of ion homeostasis. Both KCC2 isoforms are predominantly expressed in neurons of the brain and spinal cord, organs with several physiological and functional differences to the kidney. These differences are evident in the findings of Uravov and colleagues who noted that inhibition of KCC2 mRNA expression differs between neuronal and nonneuronal cells. KCC2 mRNA expression is mediated by RE-1 silencing transcription factor in nonneuronal cells, which represses the* SLC12A5* gene [39]. In neurons, however, the transcription factor early growth response 4 (Erg4) is developmentally upregulated, stimulating an increase in KCC2. This indicates fundamental differences in KCC2 expression between cell types [40]. Further research in CNS specific cell types (e.g. neuroblastoma or primary neurons) is required to determine the therapeutic implications of KCC2 expression.

In animal models of traumatic and ischaemic brain injury, KCC2 is reportedly downregulated at both the protein and mRNA levels [41–43]. Six hours after transient forebrain ischaemia, the KCC2 peptide became more abundant in the dendritic regions of pyramidal cells in the* cornu Ammonis 1 *(CA1) region of the hippocampus, which displayed no evidence of damage. Over an extended time period (48 h after stroke induction), the same cells began to degenerate in a manner that correlated with downregulation of KCC2 and heat-shock protein 72 (HS72). HS72 can exacerbate or attenuate hypothalamic neuronal death depending on its peptide expression levels and is not expressed in the mature brain under standard conditions [44]. Parvalbumin positive interneurons, which exhibit high* SLC12A5* gene expression and glutamatergic input, often survive these events even in regions of complete pyramidal cell loss [45]. This suggests that KCC2 expression is also mediated by brain health; upregulation of the cotransporter may indicate onset or previous infliction of neurological insult.

## 3. Neuronal Expression of KCC2

KCC2 is heavily expressed in the mature CNS and is rarely found in peripheral neurons and nonneuronal cells [46–48]. Upregulation of KCC2 is correlated with neuronal differentiation which occurs caudally to rostrally in the CNS [49]. In the rodent CNS, the caudal section, i.e., spinal cord and brain stem, shows little difference in KCC2 expression compared to that observed in the more mature neuron [49–51]. Conversely, rostral regions such as the hippocampus and neocortex display upregulation of* SLC12A5* mRNA from birth [49, 52].

Whilst KCC2 clearly displays region specificity within the body, these studies fail to consider variation in the cotransporter's expression between species. In rats and mice, for example, KCC2 levels remain low resulting in greater *E*_GABA_ [12]. Data collected by Dzhala et al. (2005) showed that a similar expression pattern was present in neonatal humans. Human parietal lobe autopsy specimens displayed high neuronal expression of NKCC1 and low expression of KCC2 but only before the end of the first year of life [11]. Conversely, work conducted by Sedmak et al. (2016) noted KCC2 expression begins much earlier in humans, during the mid-foetal period and increases to levels resembling adult maturity 6 months after birth [53]. Such inconsistencies may be explained by the use of only a single brain region in Dzhala's study. Alternatively, differences in maturation between humans and rodents may be responsible. Neonatal rat and mice cortices, for example, achieve a developmental stage which equates to the beginning of the third trimester of gestation in the human foetus [54, 55]. Together, these data indicate that KCC2 expression may be considered both species- and age-dependant.

KCC2 protein expression has also been associated with Ca^2+^-dependent mechanisms following neuronal damage [56–58]. Various studies have shown that KCC2 activity is heavily reduced after cleavage at the C-terminal domain by calpain proteases. Hypoxic-ischaemic encephalopathy is considered a major contributor to long-term neuronal damage with an apparent relationship between increased intracellular Ca^2+^ and neuronal damage under hypoxic conditions [59]. Calpains are Ca^2+^-dependent proteases. Perinatal mammals exhibit a high calpain/calpastatin (the inhibitor of calpain) ratio. Calpain overexpression or excessive activity has been associated with the symptoms of several neurological conditions including hypoxic ischaemia [60, 61], seizures [57], and epilepsy [62]. KCC2 upregulation is required during neuronal maturation to enhance the inhibitory properties of GABA_A_R [12, 21]. This process is, therefore, highly sensitive to excessive calpain activity causing a paucity of active KCC2. Thus, calpains may play a fundamental role in the aetiology of these diseases.

## 4. Regulation of KCC2 Activity

### 4.1. Phosphorylation

The activity and expression of KCC2 at the plasma membrane is regulated by phosphorylation. KCC2's carboxyl-domain is the target for several known kinases and is regularly phosphorylated at the serine 940 (S940) residue. Phosphorylation of S940 decreases KCC2 internalisation maintaining high KCC2 membrane expression [63]. This process is regulated by protein kinase C (PKC) which directly phosphorylates S940 resulting in greater transporter activity [63]. In contrast, dephosphorylation causes a fall in KCC2 activity mediated by a reduction in transporter stability [64]. The S940 residue and PKC activity are, therefore, key components in KCC2 regulation. Modulation of PKC activity by separate pathways, therefore, also indirectly regulates KCC2 activity and Cl^−^ homeostasis. Of note is the neuropeptide oxytocin which was found to increase KCC2 activity and support GABAergic signalling by Leonzino et al. (2016) [65]. Using PKC-inhibitors, Leonzino and colleagues prevented oxytocin mediated KCC2 upregulation suggesting a regulatory role of the neuropeptide in this process [65].

The neurotransmitter serotonin has also been reported to influence KCC2 activity. Serotonin binds and activates the receptor* 5-hydroxytryptamine type 2A* (5-HT2A) in a process that increases cell membrane KCC2 levels and subsequently restores endogenous synaptic inhibitory mechanisms in mouse models displaying injury to the spinal cord [66]. This serotonin-mediated activity is believed to be PKC-dependent given that PKC inhibitors reduced KCC2 activity [66]. Together, these results suggest S940 phosphorylation is influenced by several pathways. Given the crucial regulatory role of this residue, we can infer that the transporter's expression oscillates according to a variety of paracrine stimuli. Given the increased cell-surface density of KCC2 during S940 phosphorylation, this may be a particularly promising area of therapeutic study. Therapeutic enhancers of S940 phosphorylation may prove effective in this field especially given the recent finding that KCC2 potentiation can limit onset and severity of neuropathic seizures [67].

The dependence on C-terminal domain integrity displayed by KCC2 makes this domain a potential target for therapeutic treatments. For example, KCC2 membrane stability is heavily reduced when tyrosine residues 903 and 1087 are phosphorylated causing its subsequent trafficking to the lysosome [64]. In addition, the threonine^906^ (Thr^906^) and threonine^1007^ (Thr^1007^) residues display inhibitory characteristics when phosphorylated [68, 69]. During the neonatal period, brain localised Thr^906^ and Thr^1007^ are often phosphorylated, thereby preventing premature KCC2 activity [68, 69]. Mutants of KCC2, however, commonly show variation at these phosphorylation residues. Mutations at S932 to aspartate (S932D, mimicking phosphorylation) or T1008 to alanine (T1008A, mimicking dephosphorylation) significantly enhance KCC2 activity (up to 1.5-2-fold increase) in HEK293 cells [70]. Mutation at S940 to alanine (S940A, mimicking dephosphorylation)* in vivo* reduces KCC2 activity and enhances the effects of kainate-induced status epilepticus [36]. In contrast, Thr^906^A/Thr^1007^A double-point alanine substitution enhances KCC2 function in cell culture [67, 68, 71, 72]. Interestingly, Thr^1007^A mutations do not impact KCC2 surface expression. Preventing phosphorylation of Thr^906^ and Thr^1007^ is, however, sufficient to enhance the Cl^−^ extrusive properties of KCC2* in vivo *[67]. Such findings suggest that this is not just the result of increased KCC2 protein but rather multiple processes. The authors hypothesised that these mutations increase KCC2 affinity for Cl^−^. KCC2 Thr^906^A/ Thr^1007^A variant-carrying neurons reached Cl^−^ equilibrium at a more negative* E*_GABA_ than the wild type control. When Cl^−^ admittance is low, the increased Cl^−^ affinity displayed by these variants aids extrusion at levels beyond the wild-type threshold [67]. This increase in KCC2 function was sufficient to reduce chemoconvulsant-induced seizure activity and severity [67], suggesting that the cotransporter has therapeutic potential as a seizure limiting drug target.

Recent data provided by Friedel et al. (2015) showed the protein, With-no-lysine kinase 1 (WNK1) stimulated phosphorylation of both Thr^906^ and Thr^1007^ by means of the Kinase, Ste20-related proline alanine-rich kinase (SPAK) [69]. SPAK was phosphorylated and subsequently activated by WNK1 inhibiting KCC2 activity [69]. SPAK function and phosphorylation may also fluctuate throughout development depending on WNK1 activity [73]. Should phosphorylation of KCC2 residues Thr^906^ and Thr^1007^ occur in immature brains but fall during development, it may explain why KCC2-dependent Cl^−^ extrusion dominates in the adult CNS [69]. WNK1 is, therefore, a key regulator of KCC2 activity and a potential therapeutic target for the treatment of excitatory/inhibitory disorders.

Interestingly, Friedel et al. (2015) also found that inhibition of WNK1 dephosphorylated KCC2 at Thr^906^ and Thr^1007^ [69]. This relationship was noted in other studies suggesting a regulatory role of WNK1 in KCC2 activity. KCC2 activity assays showed the amino acid taurine significantly inhibited KCC2 via serine/threonine phosphorylation compared to control and also activated WNK1 [74]. This corroborates Friedel et al. (2015) who showed that inhibition of WNK1 increased [Cl^−^]_i_ extrusion in a KCC2-dependent manner in cultured rat hippocampal and cortical neurons [69]. Genetic studies examining changes in WNK1 activity may elucidate the aetiology of many neurological diseases.

Using the organic compound* N*-ethylmaleimide (NEM), Conway et al. (2017) increased KCC2 activity through increased S940 phosphorylation and decreased Thr^1007^ phosphorylation [72]. Interestingly, NEM was found to potentiate KCC2 activity in neurons, particularly in cells with higher pThr^1007^ levels or lower pS940 [72]. Furthermore, KCC2 mutation S932D could abolish further stimulation by NEM, whereas T1008A by another KCC2 activator, staurosporine [70]. Such findings provide valuable insight into therapeutic limitations as drugs that act to modulate KCC2 surface levels or intrinsic conformational change through phosphorylation [33, 34] would only be effective in cases of high pThr^1007^ or high pThr^1008^ and low pSer^940^ or low pSer^932^. These attributes are more common in cases of spinal cord injury. Nevertheless such drugs may be of some use in the treatment of neurological disorders. Despite this limitation, their work suggests that manipulation of Thr^1007^ phosphorylation may prove relevant to the advancement of neurological therapeutics.

An independent study identified the regulatory role of five phosphosites Ser^31^, Thr^34^, Ser^932^, Thr^999^, and Thr^1008^ using alanine and aspartine mutants [70]. Substitution of Ser^31^, Thr^34^, and Thr^999^ did not affect KCC2 activity. Ser^932^D (mimicking phosphorylation) and Thr^1008^A (mimicking dephosphorylation), however, increased transporter activity [70]. In addition, treatment with the known KCC2 activators NEM or staurosporine was ineffective in activating Ser^31^D, Thr^34^A, Ser^932^A/D, Thr^999^A, Thr^1008^A/D or Ser^31^A, Thr^31^D, Ser^932^D KCC2 variants, respectively [70]. These results demonstrated the existence of phosphosensitive sites that regulate KCC2 activities via the integration of various signalling pathways.

### 4.2. Trophic Factors

KCC2 activity is modulated by a number of trophic (growth) factors including TGF-*β*2 [75], neurotrophic factor [76], and brain-derived neurotrophic factor (BDNF) [57]. Of these, BDNF is the most well-studied modulator of KCC2 activation.

BDNF is a 27-kDa polypeptide involved in neuronal survival, differentiation, and growth [77]. Its role in KCC2 regulation was first discovered by Aguado et al. (2003) who noted that KCC2 mRNA levels increased with overexpression of the* BDNF* gene in developing neurons. This process was later found to utilise the Tropomyosin-related kinase (Trk) pathway, as deletion of the TrkB isoform decreased KCC2 mRNA [78]. These data suggest a proregulatory role of BDNF in immature neurons. In mature neurons, however, BDNF downregulates KCC2 at both the protein and RNA levels [79, 80].

Recently, Huang and colleagues noted BDNF-KCC2 regulation was injury dependent. In intact animals, BDNF downregulated membrane-bound KCC2. In animals with spinal cord injury, however, BDNF upregulated the cotransporter [81]. Reasons for these differences are not yet understood, although the authors suggested one hypothesis based on BDNF-TrKB receptor binding. This causes activation of signal pathway components such as PLC*γ*; BDNF downregulates KCC2 in the presence of PLC*γ* but upregulates it when PLC*γ* is lacking. Given that spinal cord injury has previously been found to decrease PLC*γ* expression [80], it may play a logical role in injury-dependent KCC2 regulation. Interestingly, a separate study has shown that BDNF plays a crucial role in KCC2 upregulation after seizure-induced neuronal insult [57]. Together, these studies suggest that targeting BDNF may be of therapeutic value in the treatment of diseases involving KCC2 downregulation.

### 4.3. Transcriptional and Translational Regulation

KCC2 expression is exclusive to neuronal cells, as dictated by the activity of a neuron-restrictive silencing element (NRSE) acting at the first intron of* SLC12A5* [82, 83]. A 1.4 kb promoter fragment is also implicated in KCC2 neuron expression. This was identified in a transgenic model lacking NRSE. Cells lacking NRSE showed increased levels of KCC2 expression and also expressed the active 1.4 kb promoter fragment [39]. The transcription factor Erg4 has since been found to bind to this promotor fragment and regulate KCC2 expression [40].* SLC12A5* also displays a second binding site within its promoter region known as the E-box region, which binds upstream stimulating factors (USF) 1 and 2. USF1 is negatively regulated by amyloid precursor protein (APP) which simultaneously downregulates KCC2 [84]. USF1 is, therefore, a potentially key component in the expression pattern of KCC2. Regulatory proteins such as APP and USF1 may act as biomarkers for the early identification of neurological and epileptic disease.

For successful Cl^−^ extrusion, KCC2 must be expressed at the cell surface. A further role in which APP is implicated is the stabilisation of KCC2 at the cell membrane. Direct binding of APP to KCC2 blocks phosphorylation of the tyrosine residues (903, 1087) which normally promote transporter internalisation and degradation [85]. In this way, APP acts as both a pre- and posttranslational regulator of KCC2 activity and displays strong therapeutic potential for the treatment and/or diagnosis of diseases associated with KCC2 dysfunction.

Surface expression of the KCC2 cotransporter is regulated by kainate receptors, through formation of molecular complexes between the kainate receptor subunit GluK2 and KCC2 [86, 87]. Phosphorylation of Gluk2 by PKC increases KCC2 activity, but PKC can also act directly on the cotransporter due to activation of group 1 metabotropic glutamate receptors (mGluRs). Through induction of Ca^2+^ release from internal stores, these receptors increase intracellular levels of the cation [88]. PKC is a Ca^2+^-sensitive kinase meaning its subsequent activation by group 1 mGluRs is an important component of KCC2 recorded activity. In this way, glutamatergic signalling can indirectly enhance inhibitory GABAergic signalling through increased KCC2 activity [88]. This process is implicated in maintaining equilibrium between excitatory and inhibitory signals [88]. Many neurological diseases are attributed to imbalance of these signals. This indirect mechanism of KCC2 regulation, therefore, presents a potential therapeutic pathway for drug targeting.

## 5. The Role of KCC2 in the Development of Epilepsy

The role of KCC2 mutants in epilepsy development was discovered in two separate studies conducted on patients displaying different epileptic symptoms. The first studied an Australian family suffering from febrile seizures and identified an arginine-to-histidine substitution at position 952. This missense mutation, formally named R952H, caused a substantial decrease in KCC2 membrane expression compared to the wild-type control [89]. The second, conducted by Kahle and colleagues, investigated idiopathic generalised epilepsy in a cohort of Canadian patients displaying the same mutation, c.2855G>A (R952H) [90]. Companion studies noted a significant decrease in Cl^−^ extrusion compared to control indicative of KCC2 impairment [90].

Kahle et al. (2014) also found a second KCC2 variant, R1049C, with a cysteine substitution at the 1049 position. According to* in silico* bioinformatics programmes, this mutation is predicted to possess pathogenic properties that correlate with KCC2 dysfunction [90]. In accordance with the findings of Puskarjov et al. (2014), Kahle and colleagues showed that R952H mutants had a significantly lower level of KCC2 expressed at the cell surface. In R1049C mutants, however, KCC2 levels were not noticeably different to control [89, 90]. R1049C reduced KCC2 efficacy for Cl^−^ extrusion, resulting in higher basal [Cl^−^]_i_ levels and membrane depolarisation at the previously inhibitory synapse [90]. Both variants also displayed a significant (>50%) decrease in S940 phosphorylation. Thus, both R952H and R1049C C-terminal mutations reduce KCC2 activity. This, in part, may be due to a decrease in stimulatory S940 phosphorylation [90]. Alternatively, interaction of these variants with the ISO domain (a unique 15 amino acid region on the KCC2 C-terminal domain) which has previously been identified as a vital component to KCC2 isotonic activity may cause the observed reduction in KCC2 function [91].

More recently, Stödberg and colleagues identified an autosomal recessive heterozygous loss-of-function mutation in the* SLC12A5* gene in children from two separate families [92]. In both families, two children developed clinical features of epilepsy of infancy with migrating focal seizures (EIMFS). All mutated residues were of KCC2b lineage: L288H, L403P, and G528D. Of the four children examined, two had compound heterozygous mutations, c.1208T>C (p.L403P) and c.1583G>A (p.G528D), whilst the others had homozygous missense mutations, c.863T>A (p.L288H) [92]. L403P and G528D mutants displayed complete loss of KCC2-mediated Cl^−^ extrusion, whilst the homozygous L403P mutant had reduced surface expression and glycosylation leading to partial loss of function [92]. Their data further contributes to the growing evidence that disruption of KCC2 activity is implicated in epilepsy. Research into additional mutations affecting* SLC12A5 *may provide novel insight into the individual application of antiepileptic strategies.

There are, however, limitations to the data collected here that cannot be overlooked. All variants described in these studies were only identified through examination of the* SLC12A5* gene sequence. The need for whole genome sequencing intervention to identify other variants or alleles not encoded by* SLC12A5 *but that augment KCC2 activity was raised by these studies [89, 90, 92].

Another study conducted by Saitsu et al. (2016) also identified four previously undiscovered KCC2 variants that resulted in EIMFS [93]. In a sample of ten sporadic and one familial case of EIMFS, whole exome sequencing identified compound heterozygous* SLC12A5 *variants in two families: c.279 + 1G > C causing skipping of exon 3 in the transcript (p.E50_Q93del), c.572 C >T (p.A191V) in two siblings, and c.967T > C (p.S323P) and c.1243 A > G (p.M415V) in another individual. Another patient with migrating multifocal seizures carrying compound heterozygous mutations, c.953G>C (p.W318S) and c.2242_2244delTCC (p.S748del), was also identified from whole exome sequencing data of 526 patients and targeting of the* SLC12A5* sequence from a cohort of 141 patients with infantile epilepsy [93]. Gramicidin-perforated patch-clamp analysis identified a reduction in Cl^−^ extrusion of E50_Q93del and M415V mutants, with mildly impaired function of A191V and S323P mutants. Membrane expression of these KCC2 variants did not differ from control. Heterologous expression of two KCC2 variants, however, mimicking the patients' status, showed significantly higher [Cl^−^]_i_ levels than wild-type KCC2 but lower levels compared to the group lacking KCC2 [93]. These findings indicate that even partial disruption to neuronal Cl^−^ extrusion, mediated by two impaired variants of* SLC12A5*, causes EIMFS.

Since these discoveries, gene panel sequencing of an EIMFS patient from an unrelated family found a compound heterozygous constellation of variants in* SLC12A5* consisting of a maternally inherited p.Ser399Leu and a* de novo* p.Arg880Leu mutation in human KCC2b [93]. Such mutations may be pathogenic.

## 6. KCC2 in Neurodevelopmental Disorders

KCC2's C-terminal domain is encoded at the 3' end of the* SLC12A5* gene [90]. Recently, Merner et al. (2015) investigated KCC2 regulatory variation using Sanger sequencing to investigate the coding nucleotides 21-25 of the* SLC12A5* gene [94]. The authors screened a total of 427 autism spectrum disorder (ASD), 143 schizophrenic, and 190 intellectual disability cases [94]. R952H and R1049C were among the mutations found in ASD cases. Interestingly, R952H was also implicated in schizophrenia, suggesting overlap between these disorders. Different phenotypic outcomes from R952H mutation (i.e., which disease the patient has) are likely dependent on other allele interactions.

Thorough understanding of how risk alleles contribute to disease is not yet established. In polygenic disease models, causality is never attributed to just one variant [95]. Merner showed that patients with ASD carried rare KCC2 variants that affected CpG sites [94]. CpG sites are prone to methylation, a process that can alter the expression pattern of the gene [96]. Variation in* SLC12A5* expression in patients with ASD may, therefore, be the consequence of epigenetic interactions, which represent a potentially valuable focus for future research.

## 7. KCC2 in Neuropathic Pain

Neuropathic pain (NP) is characterised by spontaneous pain sensations and tactile allodynia. The system of pain detection requires a balance of excitatory and inhibitory signals. When this balance is disrupted either through injury or psychogenic insult, it can lead to NP. In both the spinal cord and dorsal horn, synaptic transmission patterns vary between NP models [97, 98]. This pain has been attributed to dysfunctional inhibitory mechanisms in the spinal cord. In fact, pharmacological disruption of synaptic inhibition within the dorsal horn induces symptoms commonly attributed to NP [99]. Reduction of the Cl^−^ gradient across the neuronal membrane has since been identified as the cause of NP initiated by peripheral nerve injury [100]. This is the result of downregulation of KCC2. During NP pathogenesis, an array of cellular mechanisms converge causing a reduction in KCC2 expression and function and increase in neuronal [Cl^−^]_i_ [100]. The need to identify cellular mechanisms that increase KCC2 activity during neuropathic episodes is, therefore, crucial to the advancement of therapeutics in this field.

Increasing KCC2 activity presents a very prudent area of research [101–103]; the ability to restore normal inhibitory function in neurological conditions associated with impaired Cl^−^ transport may prove to be an effective therapeutic strategy. High-throughput screening assays have now identified KCC2 activators that reduce [Cl^−^]_i_. Gagnon et al. (2013) optimised a first-in-class arylmethylidine family of compounds (CLP257) to lower [Cl^−^]_i_ [104]. CLP257 rescued KCC2 plasma expression, renormalised stimulated recall responses in spinal nociceptive pathways sensitized after nerve injury, and reduced hypersensitivity of NP rat models [104]. The results of Cardarelli et al. (2017), displaying CLP257 as a direct KCC2 activator, were not replicable [105] but do reveal the compounds' ability to potentiate GABA_A_R activity [105]. Furthermore, GABA_A_R-dependent synaptic inhibition by KCC2 antagonist, gabazine could actually tune KCC2 activity via the Cl^−^-sensitive WNK1 kinase [106]. Oral treatment of the CLP257 prodrug equivalent, CLP290, showed similar efficacy to their control of pregabalin, a drug commonly used in the treatment of epilepsy and anxiety [104, 107]. Side effects of pregabalin include dizziness and sedation causing motor function disturbance [107]. Such side effects were not present during treatment with CLP290 [104]. These results highlight KCC2 as a plausible target for NP drug therapy and may provide further insight into the treatment of other neurological disorders.

## 8. Therapeutic Potential of KCC2

KCC2's interaction with Cl^−^ importer GABA_A_R makes it a potential target for the treatment of several neurological diseases. Currently, phenobarbital (PB), a barbiturate that delays the closing of GABA_A_R, is the most common first-line drug used for the treatment of seizures [108]. Hypoxic-ischaemic encephalopathy is a major contributor to the onset of neonatal seizures, with over 50% of patients displaying electrographic seizures even after treatment with PB [109]. Interruption to the expression and/or function of either KCC2 or NKCC1 affects the antiseizure efficacy of GABA_A_R agonists [110]. The higher [Cl^−^]_i_ within immature neurons potentially contributes to resistance to pharmacological first-line antiseizure GABA_A_R agonists in the immature brain [111].

Recently, a translational model for age-dependent PB-resistant seizures was developed by Kang et al. (2015) [112]. Using a permanent unilateral carotid-ligation model of neonatal ischaemic-seizures in CD-1 pups, the authors investigated the ability of the NKCC1 antagonist bumetanide to rescue PB-resistance. Bumetanide failed to rescue PB as an antiseizure therapeutic [112]. A number of preclinical models show that the severity of seizure and mechanism of damage can influence the efficacy of antiseizure drugs and alter cotransporter expression [113–116]. Kharod et al. (2018) noted model-specific insults modulated both expression and function of the NKCC1 and KCC2 cotransporters. Using a pentylenetetrazol-induced seizure model, they identified a significant upregulation of KCC2. In contrast, ischaemia-induced seizures significantly downregulated KCC2 [117]. These data combined reveal KCC2 expression to be insult specific and may explain why some anticonvulsant therapies display variable efficacy during first-line treatment.

Activation of the Trk isoform TrkB has been shown to induce phosphorylation of phospholipase C*-γ*1 which is linked to the downregulation of KCC2 and development of epilepsy [118, 119]. Carter et al. (2018) showed that TrkB antagonist, ANA12, increases the efficacy of PB in CD1 mice at doses as low as 2.5 mg/kg. ANA12 also rescued KCC2 expression after postnatal ischaemia [120]. Unlike current clinical antagonists (e.g. bumetanide, furosemide), ANA12 is capable of passing through the blood-brain-barrier [121], allowing it to have greater therapeutic impact on KCC2 activity as this has previously been a limiting factor for treatments [122]. ANA12 may, therefore, have therapeutic benefit by preventing downregulation of KCC2, thus maintaining low [Cl^−^]_i_.

## 9. Conclusion

KCC2 is a key player in the maintenance of neuronal Cl^−^ homeostasis. A plethora of studies identify KCC2 dysfunction and misregulation as a key component in the development and onset of many neurological diseases. KCC2 is a strong candidate for therapeutic targeting and should be further considered by pharmaceutical investors. It should be noted that the majority of these findings are not made in human neuronal cell lines and are, therefore, limited in their ability to determine the immediate effects of targeting KCC2. Despite this, the data collected from human participants indicates that there is a place for KCC2 pharmaceuticals in the treatment of epilepsy. Continued research in human neuronal cell types may reveal more opportunities for drug development.
